# Stroke and thrombolysis: an old disease with a new approach

**DOI:** 10.1186/cc9738

**Published:** 2011-03-11

**Authors:** N Catorze, L Pessoa, M Abu Hazima, F Carrilho

**Affiliations:** 1 Centro Hospitalar Médio Tejo, Abrantes, Portugal

## Introduction

The incidence of stroke doubles for every decade after 45 years of age, and 70% of these events occur in the over 65 s. A rational approach with a thrombolysis protocol can diminish this clinical and social burden.

## Methods

In the past 20 months all patients with acute stroke were referred to ICU staff for evaluation and compliance to effective thrombolysis until 4.30 hours from the onset of symptoms. All clinicians were advised and triage in the ED was adapted using NIHSS.

## Results

In this period 152 patients were evaluated and 34 (22.4%) were eligible for reperfusion treatment. Men were more prevalent than women (70.6 vs. 29.4%) and age was distributed between 29 and 82 years. Risk factors were equally distributed (Table 1). Twenty-nine patients (88%) received thrombolysis within 3 hours of symptoms onset and 19 (63%) got better NIHSS after treatment. Eleven patients (37%) never recovered. Five out of 34 patients (12%) were treated in the 3 to 4.30 hours window and three received benefit. All deaths were related to ischemia progression. Table 2 presents complications during the ICU stay.

**Table 1:** Stroke risk factors

Factor	*n*
High blood pressure	25
AF	4
>Lipids	6
Diabetes	4
>BMI	6
Smoke	10

**Table 2:** Complications during the ICU stay

Complication	*n*
Bradycardia	8
Pneumonia	5
Hemorrhage	4
Death	4

## Conclusions

The clinicians' compliance and patients' reference to dedicated teams (stroke teams) resulted in the treatment of 22.4% of observed patients (1 to 11% in the literature). Some complications could be avoided with simple measures. This protocol should continue and should be emphasized.
